# Robotic and Video-Assisted Thoracic Surgery for Early-Stage Lung Cancer: Comparison of Long-Term Pain at a Single Centre

**DOI:** 10.3390/jcm11041108

**Published:** 2022-02-19

**Authors:** Alberto Testori, Veronica Maria Giudici, Emanuele Voulaz, Marco Alloisio, Edoardo Bottoni

**Affiliations:** 1 Division of Thoracic Surgery, Humanitas Clinical and Research Center, Via Manzoni 56, Rozzano, 20089 Milan, Italy; alberto.testori@humanitas.it (A.T.); veronica.giudici@humanitas.it (V.M.G.); marco.alloisio@hunimed.eu (M.A.); edoardo.bottoni@humanitas.it (E.B.); 2 Department of Biomedical Sciences, Humanitas University, Rozzano, 20089 Milan, Italy

**Keywords:** lung cancer, post-operatory pain, mini-invasive treatment

## Abstract

Backgrounds: Since the application of mini-invasive surgery to pulmonary lobectomy, various studies confirmed the feasibility and the safety of the technique, with equivalent oncological standards. However, there are no studies that compare long-term postoperative pain in minimally invasive thoracic surgery. Methods: Between 1 January 2019 and 28 February 2020, we analysed pain scores at 2 weeks, 3 months, 6 months, and 1 year after the operation, where 50 patients underwent a VATS lobectomy and 50 underwent a RATS lobectomy. Pain scores are obtained through a telephone questionnaire, according to a Numerical Rating Scale (NRS). Results: The medias of the NRS scores, at 2 weeks, 3 months, 6 months, and 1 year after the operation were similar in both groups. Group I was composed of 50 patients who underwent a video-assisted lobectomy, while Group II was composed of 50 patients who underwent a robotic-assisted lobectomy. Two weeks after surgery Group I had a NRS value of 2.96 and in Group II it was 2.86; three months after in Group I the value was 2.16 and in Group II it was 2.06; six months after Group I ‘s value was 1.62 and Group II’s was 1.56; one year after in Group I the value was 1.30 and in the Group II was 1.24. For each time interval, no statistically significant differences were found (*p* > 0.05). Conclusions: In our analysis, RATS and VATS did not have significant differences in post-operative and long-term pain.

## 1. Introduction

Mini-invasive surgical treatment is considered the approach of choice in clinical stage I of lung cancer resectable in lobectomy [1]. Since the introduction of video assisted thoracic surgery (VATS) in pulmonary lobectomies, various studies confirmed the feasibility and the safety of the technique, along with equivalent oncological standards. When robotic-assisted thoracic surgery (RATS) lobectomies resulted as a valid approach, numerous papers stated the oncological efficacy and the reduction in terms of postoperative pain, impairment in pulmonary function, chest tube duration, and hospital stay compared to thoracotomy [2,3]. Veronesi et al. have not found any statistical differences between RATS and VATS in terms of conversion rate and postoperative complications [4,5]. Comparing minimally invasive techniques, some authors highlighted some benefits of RATS compared to VATS, such as improved ergonomics, three-dimensional optics, wristed instrument motions and a shorter learning phase, while in contrast they also reported a longer operative time and a higher cost. Most literature compares VATS and RATS in terms of postoperative outcomes of pain, duration of air leak, duration of chest tube drainage, hospital length of stay, oncological outcomes as lymph node dissection and survival, and, finally, financial outcomes as cost-effectiveness [5,6].

This study analyses long-term postoperative pain at 2 weeks, 3 months, 6 months, and 1 year of 50 VATS thoracic surgery lobectomies to 50 RATS lobectomies in a single centre.

All patients agreed to provide their medical information and signed informed consent, approved by the ethics committee.

## 2. Materials and Methods

In our centre, all clinical stage I of non-small lung cancer (NSCLC) were considered eligible for a minimally invasive lobectomy. We included in our analysis all patients who underwent a video-assisted or a robotic-assisted lobectomy during 2019 for a clinical stage I of NSCLC. We analysed pain scores at 2 weeks, 3 months, 6 months, and 1 year after the operation of 50 patients who underwent a VATS lobectomy and 50 of a RATS lobectomy.

Any other type of lung resection, such as wedge, segmentectomies, bi-lobectomies, or pneumonectomies, were excluded from our study. In our analysis we included only those procedures that were comparable to each other. In our centre wedge resections, a minimally invasive technique, were performed in VATS and not in the robotic technique due to the excessive costs.

Data were retrospectively collected from patient files and through a telephone questionnaire. Pain scores were obtained according to the Numerical Rating Scale (NRS, 1–10).

**Figure 1:**
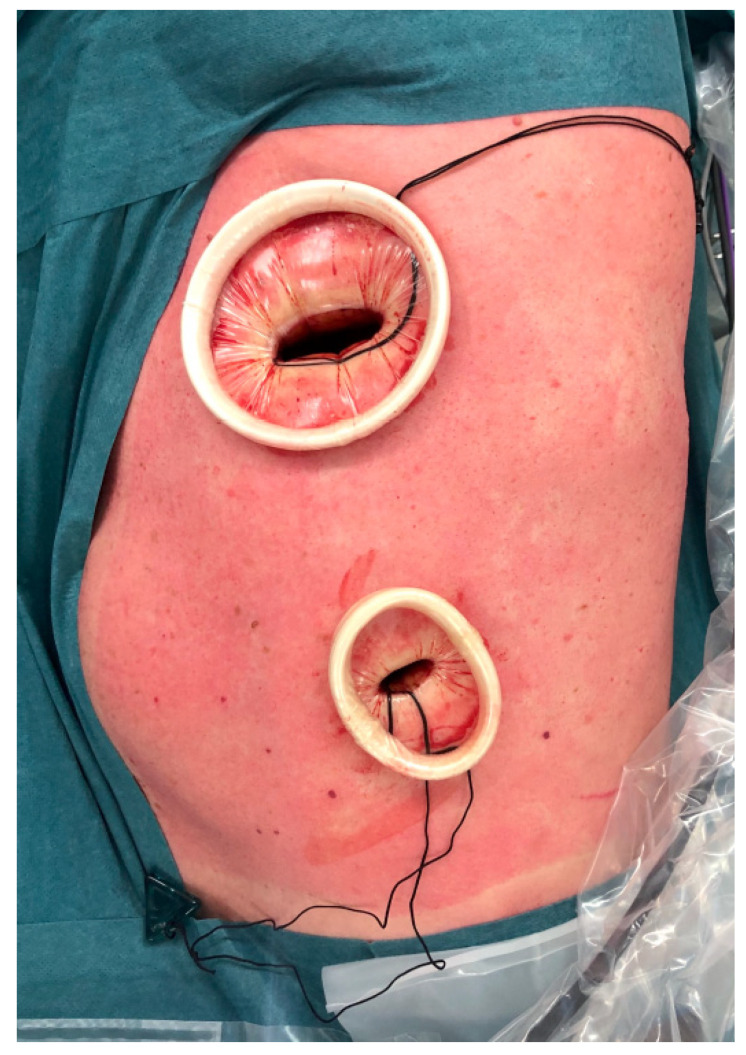
VATS approach with two incisions. A utility incision performed in the 4th intercostal space and a video-port at the 7th intercostal space.

**Figure 2:**
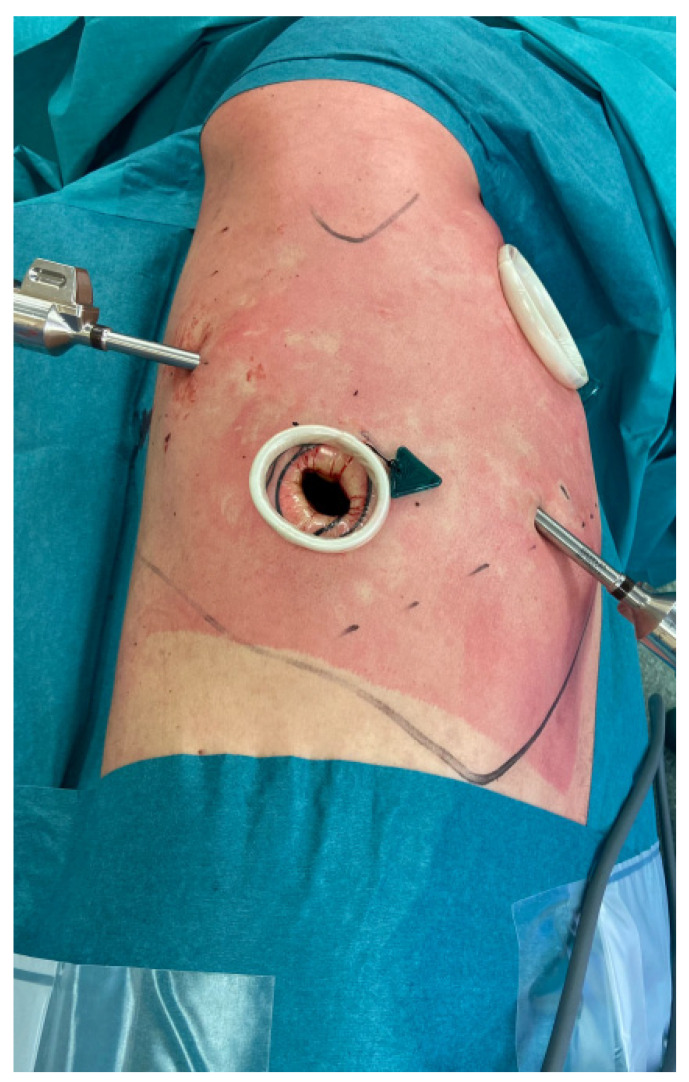
Incisions of a Right Upper Lobectomy performed in RATS. An utility incision at 4th intercostal space and three additional ports.

All surgery was performed in lateral decubitus position with single lung ventilation and general anaesthesia. VATS lobectomies were performed through two incisions: a 15-mm video-port in the 7th or 8th intercostal space in the mid-axillary line where the 30° camera was placed through a soft tissue retractor (Alexis, xs, Applied Medical, California) and a 30-mm utility incision was performed in the anterior axillary line in the 4th or 5th intercostal space and another soft tissue retractor (Alexis, s, Applied Medical) was placed (Figure 1). RATS lobectomies were performed using DaVinci robot surgery system type Xi (Intuitive, California) with a four arms approach. It was carried out through a utility incision at the fourth intercostal space and three additional ports without CO_2_ use, according to the Park-Veronesi technique. (Figure 2).

The linear stapler device was used to divide the hilum elements and the fissures. Nodal dissection was performed similar in all surgery with the removal of nodes in three different mediastinal lymph node stations.

Statistical analysis was performed using Microsoft Excel^®^ vers. 16.58 and IBM^®^ SPSS Statistics Software.

## 3. Results

Between 1 January 2019 and 28 February 2020, we enrolled 50 patients who underwent to a video-assisted lobectomy, named Group I, and 50 robotic-assisted lobectomy named Group II. The majority of patients were male (57%), and the median age was 68 years.

The two groups under analysis presented overlapping characteristics. The average age in Group I, the VATS group, was 69.1, while in Group II, the RATS group, it was 67.1. No statistically significant differences were found (*p* > 0.05). The percentage of men between the two groups differed slightly: 68% in Group I and 64% in Group II.

The medians of the NRS scores, at 2 weeks, 3 months, 6 months, and 1 year after the operation were similar in the groups.

**Figure 3:**
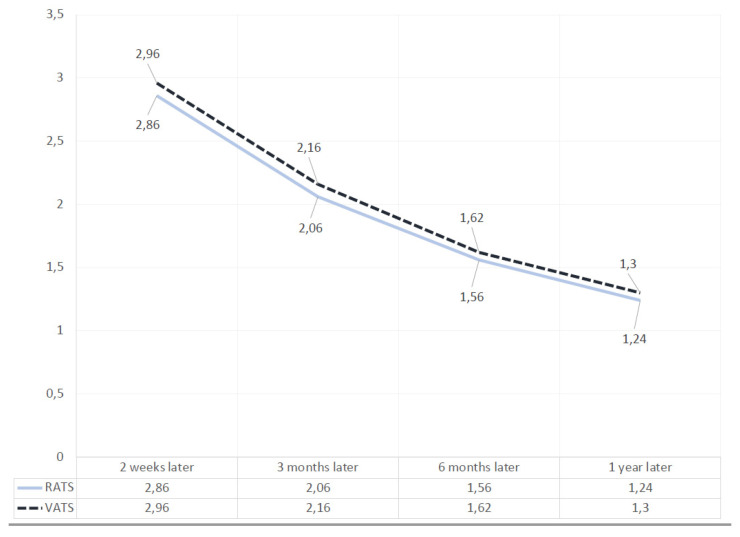
Table shows the trend of scores of the VATS and RATS lobectomies at 2 weeks, 3 months, 6 months, and 1 year after surgery.

Two weeks later, in the video-assisted lobectomies group, the NRS value was 2.96 (with a standard deviation of 0.83), while in the robotic group it was 2.86 (a standard deviation of 1.05); 3 months later in Group I the value was 2.16 (standard deviation of 0.65) and in Group II was 2.06 (standard deviation: 0.87); 6 months later in Group I the NRS value was 1.62 (standard deviation of 0.64), while in Group II was 1.56 (standard deviation of 0.67); 1 year later in Group I the value was 1.30 (standard deviation of 0.54) and in Group II was 1.24 (standard deviation of 0.47). For each time interval, no statistically significant differences were found (*p* > 0.05) (Figure 3).

The percentage of patients of Group I who continued to assume analgesic therapy was 8%, while in Group II this was 6%; even in this case the difference was not significant.

## 4. Comment

Various papers and subsequent analyses and meta-analyses suggested that pain would be an interesting surgical parameter to evaluate, although its multifaceted nature makes it a difficult outcome to measure.

Numerous scales are used to analyze pain during post-operatories days, such as Verbal Scale and Visual Analogue Scale. Despite this, we have chosen a Numerical Rating Scale to determine postoperative pain in lung surgery, due to ease of use and the convenience of comparing the values. The NRS has good sensitivity and generates data that can be easily statistically analyzed.

In our analysis, mean pain scores remained lower than 3 in both groups, in every time interval considered (2 weeks, 3 months, 6 months, and 1 year) with no significant difference between them; According to Myles et al. [7] this is indicative of good post-operatory pain control.

Various studies analyzed postoperative pain after lung surgery conducted with different techniques. Cerfolio et al. estimate post-operatory pain of 486 patients through a quality of life with a 12-item Short Form Health Survey. After an analysis of 168 RATS and 318 open pulmonary lobectomies, the authors found that pain scores for those patients who underwent a RATS procedure were significantly lower, with a value of 2.5 versus 4.4 [8]. Bendixen et al. performed a blind randomized controlled trial to analyze the post-operative pain after VATS and thoracotomy in 206 patients with a clinical stage I of lung cancer. During the period of observation, consisting of a 52-week follow-up, episodes of moderate-to-severe pain, classified with a NRS higher than 3, were significantly less frequent after VATS lobectomies [9].

An aspect whose relevance remains to be defined is whether the type of VATS approach can assume a different role in terms of pain; in fact, we assisted to a trend of using fewer ports for VATS in lung surgery to minimize the trauma to the chest [10].

All these analyses compared lobectomies performed through thoracotomy to VATS or RATS lobectomy.

According to Van der Ploeg et al., there are not any differences in terms of pain between open thoracotomy, video-assisted thoracic surgery, and robot-assisted thoracic surgery, as in our analyses [11].

Various comparative analysis of costs was performed. The first was conducted by Park et al. considering 368 patients, where they estimated the additional cost. RATS lobectomies are reported as more expensive than VATS, adding on average $3981 [12]. In 2018, Novellis et al. estimated that the cost of robotic surgery was around 13.5% higher than for VATS and open surgery; however, the robotic approach was associated with a profit margin for the hospital of about 18% [13].

However, 3D camera and robotic-specific instruments, which increase surgical precision, represented the principal advantages of these technique. Currently, new generation robotic systems are implemented. A da Vinci system that employs multi-jointed instruments via a single port has been developed [14].

The lack of the performance of the sample size calculation prior to the initiation of the study, represent the principal limitation of our work. We will continue to analyse our operating cases to implement the power of the study.

In conclusion, this study describes post-operatory long-term pain outcomes of the two mini-invasive approaches for clinical stage I of non-small lung cancer, VATS and RATS, performed in a single center. In our experience, RATS and VATS did not have significant differences in postoperative and long-term pain.

## References

[1] Postmus P.E., Kerr K.M., Oudkerk M., On behalf of the ESMO Guidelines Committee (2017). Early and locally advanced non-small-cell lung cancer (NSCLC): ESMO Clinical Practice Guidelines for diagnosis, treatment and follow-up. Ann. Oncol..

[2] Yan T.D., Black D., Bannon P.G., McCaughan B.C. (2009). Systematic review and meta-analysis of randomized and nonrandomized trials on safety and efficacy of video-assisted thoracic surgery lobectomy for early-stage non-small-cell lung cancer. J. Clin. Oncol..

[3] Wei S., Chen M., Chen N., Liu L. (2017). Feasibility and safety of robot-assisted thoracic surgery for lung lobectomy in patients with non-small cell lung cancer: A systematic review and meta-analysis. World J. Surg. Oncol..

[4] Veronesi G., Abbas A.E.-S., Muriana P., Lembo R., Bottoni E., Perroni G., Testori A., Dieci E., Bakhos C.T., Car S. (2021). Perioperative Outcome of Robotic Approach Versus Manual Videothoracoscopic Major Resection in Patients Affected by Early Lung Cancer: Results of a Randomized Multicentric Study (ROMAN Study). Front. Oncol..

[5] Novellis P., Maisonneuve P., Dieci E., Voulaz E., Bottoni E., Di Stefano S., Solinas M., Testori A., Cariboni U., Alloisio M. (2021). Quality of Life, Postoperative Pain, and Lymph Node Dissection in a Robotic Approach Compared to VATS and OPEN for Early Stage Lung Cancer. J. Clin. Med..

[6] Wei B., D’Amico T.A. (2014). Thoracoscopic versus robotic approaches: Advantages and disadvantages. Thorac. Surg. Clin..

[7] Myles P.S., Myles D.B., Galagher W., Boyd D., Chew C., MacDonald N., Dennis A. (2017). Measuring acute postoperative pain using the visual analog scale: The minimal clinically important difference and patient acceptable symptom state. Br. J. Anaesth..

[8] Cerfolio R.J., Bryant A.S. (2011). Optimal care of patients with non- small cell lung cancer reduces perioperative morbidity. J. Thorac. Cardiovasc. Surg..

[9] Bendixen M., Jorgensen O.D., Kronborg C., Andersen C., Licht P.B. (2016). Postoperative pain and quality of life after lobectomy via video-assisted thoracoscopic surgery or anterolateral thoracotomy for early stage lung cancer: A randomised controlled trial. Lancet Oncol..

[10] Tosi D., Nosotti M., Bonitta G., Mazzucco A., Righi I., Mendogni P., Rosso L., Palleschi A., Rocco G., Italian VATS Group (2019). Uniportal and three-portal video-assisted thoracic surgery lobectomy: Analysis of the Italian video-assisted thoracic surgery group database. Interact. Cardiovasc. Thorac. Surg..

[11] van der Ploeg A.P.T., Ayez N., Akkersdijk G.P., van Rossem C.C., de Rooij P.D. (2020). Postoperative pain after lobectomy: Robot-assisted, video-assisted and open thoracic surgery. J. Robot. Surg..

[12] Park B.J., Flores R.M. (2008). Cost comparison of robotic, video-assisted thoracic surgery and thoracotomy approaches to pulmonary lobectomy. Thorac. Surg. Clin..

[13] Novellis P., Bottoni E., Voulaz E., Cariboni U., Testori A., Bertolaccini L., Giordano L., Dieci E., Granato L., Vanni E. (2018). Robotic surgery, video-assisted thoracic surgery, and open surgery for early stage lung cancer: Comparison of costs and outcomes at a single institute. J. Thorac. Dis..

[14] Suda T. (2017). Transition from video-assisted thoracic surgery to robotic pulmonary surgery. J. Vis. Surg..

